# Autologous plasma versus fetal calf serum as a supplement for the culture of neutrophils

**DOI:** 10.1186/s13104-020-4902-z

**Published:** 2020-01-22

**Authors:** Razieh Alipour, Alimohammad Fatemi, Fereshteh Alsahebfosul, Alireza Andalib, Abbasali Pourazar

**Affiliations:** 1 Immunology Department, Medical School, Isfahan University of Medical Sciences, Hezar Jerib Street, Isfahan, Iran; 2 Rheumatology Section, Department of Internal Medicine, School of Medicine and Acquired Immunodeficiency Research Center, Isfahan University of Medical Sciences, Hezar Jerib Street, Isfahan, Iran

**Keywords:** Fetal calf serum, Autologous plasma, Apoptosis, Granulocytes

## Abstract

**Objective:**

Currently, the replacement of fetal calf serum (FCS) by a more suitable alternative is a sought aim in the field of tissue and cell culture research. Autologous plasma (AP) and especially autologous serum (AS) have been shown to be effective substitutes of FCS in culture media for some of the cell types. Nevertheless, there is no comparative data on the most appropriate supplement for cell media in neutrophil studies, it is now unclear whether AP have a relatively equal, superior or inferior performance to FCS in neutrophil cell culture. In the present study, human blood neutrophils were isolated and cultured in FCS- or AP-supplemented medium. After 12, 36 and 60 h of incubation, cell viability, oxidative burst and CD11b expression were determined by flow cytometry.

**Results:**

Compared to the culture of neutrophils in FCS 10% medium, the culture of neutrophils in a medium with AP 10% could prolong their life span without affecting their function. The findings introduce AP as a better supplement for human neutrophil cell culture than FCS and propose a simple and economical procedure for neutrophil isolation and culture.

## Introduction

“Fetal calf serum” (FCS), or “fetal bovine serum” (FBS) has been using in almost every cell culture settings for years. But the use of FCS is associated with several complications [1]. The animal welfare and the likely transmission of bovine pathogens to human are two serious concerns in the field [2, 3]. The variable composition of FCS from batch to batch causes unreproducible results in research studies [4]. Furthermore, FCS in culture media exposes cells of none-bovine origins to xenogeneic proteins, which may cause to inferior functions of the cells [5]. The limited availability beside the ever-increasing demands for FCS have resulted in the unreasonable augmentation of the price and the entrance of fake products of FCS to the market [1, 6].

Different autologous/heterologous blood-derived products as alternatives to FCS have been investigated and shown promising results, too [7, 8]. These investigations have focused on adherent cell cultures [9, 10], and blood leukocytes have been ignored. But leukocytes—including neutrophils, especially in recent years—have contributed an indispensable portion of cell culture systems.

Autologous plasma (AP) and autologous serum (AS) have been introduced as substitutes of FCS in culture media which avoid many problems related to using of FCS [11]. Reported results on the replacement of FCS by AP and AS in different cell culture settings are not entirely compatible [12, 13].

While some researchers have been cultivated neutrophils with AP or AS in culture media, the potential changes in neutrophil biology and behavior by changing the culture media supplements have not been investigated yet. To find better supplementation, we compared the cell viability and functionality between human neutrophils cultured in AP- or FCS-supplemented media.

## Main text

### Methods

#### Sample collection

Blood was collected from 32 healthy volunteers (Additional file 1: Table S1) in tubes containing EDTA-ACD (acid citrate dextrose). The samples were centrifuged (250*g*, 18 ℃, 15 min) to separate platelet-rich plasma (PRP), the rest of the blood was diluted by normal saline (sterile, LPS-free). The PRP was spun (5000*g*, 4 ℃, 20 min) and the upper AS was collected and refrigerated until the use in the cultures.

#### Neutrophil isolation

After red blood cells (RBCs) sedimentation by dextran, the sample was decanted onto a 2-layered discontinuous density gradient of Percoll (86 and 55%) and centrifuged (480*g*, 17 min, 18 ℃, brake off). After centrifugation, the distinct mononuclear cells (on the Percoll 55%) and granulocytes (on the Percoll 86%) were removed separately. The neutrophils were washed and suspended in RPMI medium (Gibco).

For five of the samples, neutrophil isolation was performed by Percoll gradient (as above) as well as by Ficoll (Biosera) gradient centrifugation (25 min, 750*g*, 18 ^○^C, brake off), followed by RBC lysis using hypo-osmotic shock.

The initial cell viability was evaluated by Trypan blue. The viability had to be ≥ 98% or the experiment would not be continued. In some cases, the viability obtained by Trypan blue was checked and confirmed by flow cytometry.

#### Cell culture

To minimize the effect of variations in FCS/FBS products, we combined equal volumes of six product of FCS/FBS procured from different venders or lots and prepare a FCS/FBS mixture (one FCS product and two FBS products from Gibco plus two FCS products and one FBS product from Sigma). The mixture was used to supplement FCS cultures.

Neutrophils were cultured (density 5 × 10^5^ cell/ml) in RPMI, which was supplemented by AP 10% or FCS 10% (the mentioned mixture), at 37 °C, CO_2_ 5%, 90% humidity, for different times (12 h, 36 h and 60 h).

#### Cell viability/apoptosis measurement

After the designated culture times, neutrophils were harvested, washed and resuspended in RPMI at 1 × 10^6^ cell/ml concentration. Two aliquots of 200 µl were taken for further (CD11b and oxidative burst) analyses. The rest of the cells were stained using an Annexin-V-FITC Apoptosis detection kit (eBioscience) as per the manufacturer’s protocol and analyzed by flow cytometry.

#### CD11b expression assay

An aliquot of 2 × 10^5^ neutrophils was stimulated with 100 ng/ml of endotoxin (LPS from *Escherichia coli*, serotype 0111: B4, Sigma) at 37 °C, CO_2_ 5% for 30 min. Thereafter, the samples were stained with FITC anti-human CD11b mAb (Biolegend) or isotype control antibody (20 min at RT) and then run on flow cytometer.

#### Measurement of oxidative burst

2 × 10^5^ neutrophils were divided equally as experimental and negative samples, activated (or not for negative sample) by cell activation cocktail (Biolegend) for 20 min (37 °C, CO_2_ 5%), then dihydrorhodamine 123 (Santa Cruz) was added (final concentration of 1 µM) and re-incubated for another 20 min. Then, the cells were placed into an ice bath (10 min), then washed and suspended in phosphate buffer saline containing formaldehyde 0.5% and analyzed by flow cytometry.

Flow cytometry was performed using a FACSCalibur flow cytometer (BD). Data were analyzed by FlowJo software version X.

### Statistical analysis

Statistical comparisons were estimated using repeated measurements analysis of variance (ANOVA), using IBM SPSS-25. The results are expressed as mean ± standard error of the mean (SEM). Differences were considered significant for P < 0.05.

## Results

### Neutrophil purity

**Fig. 1:**
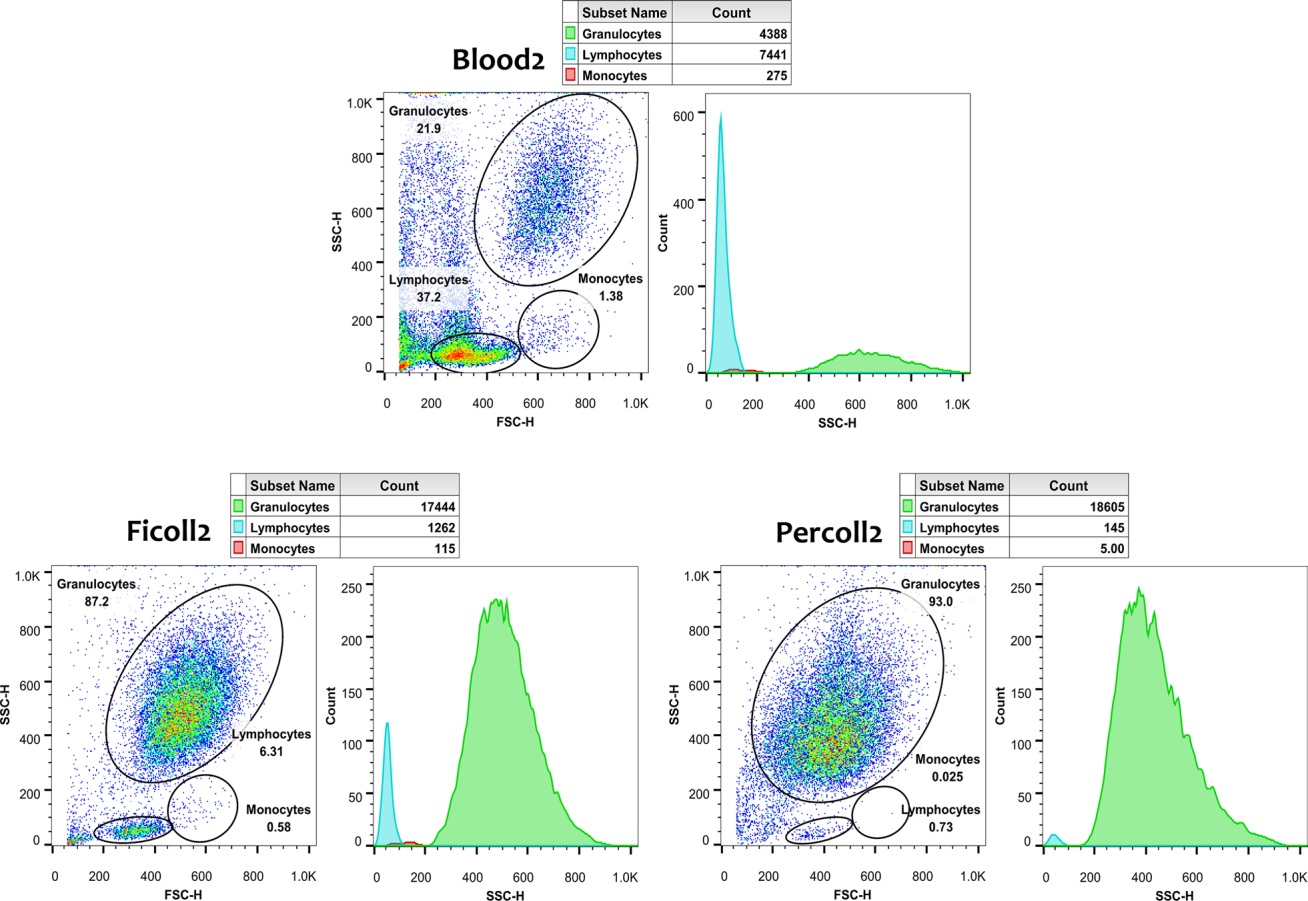
The purity of neutrophils isolated by two method. A blood sample after RBC sedimentation (upper) and then after Ficoll (lower left) and Percoll centrifugation (lower right). The mean percent of granulocytes was 98.769 ± 0.416 and 96.240 ± 1.103 of viable leukocytes for Percoll and Ficoll, respectively (n = 5)

The granulocytes were located on forward scatter (FSC)/side scatter (SSC) plots and suitable gates were set around them and also around lymphocytes and monocytes. The corresponding SSC histograms were used to identify the number of cells in each gate. Lymphocyte and monocyte contamination was less in the neutrophil population obtained by Percoll density gradient (Fig. 1).

### Neutrophil viability and apoptosis

**Fig. 2:**
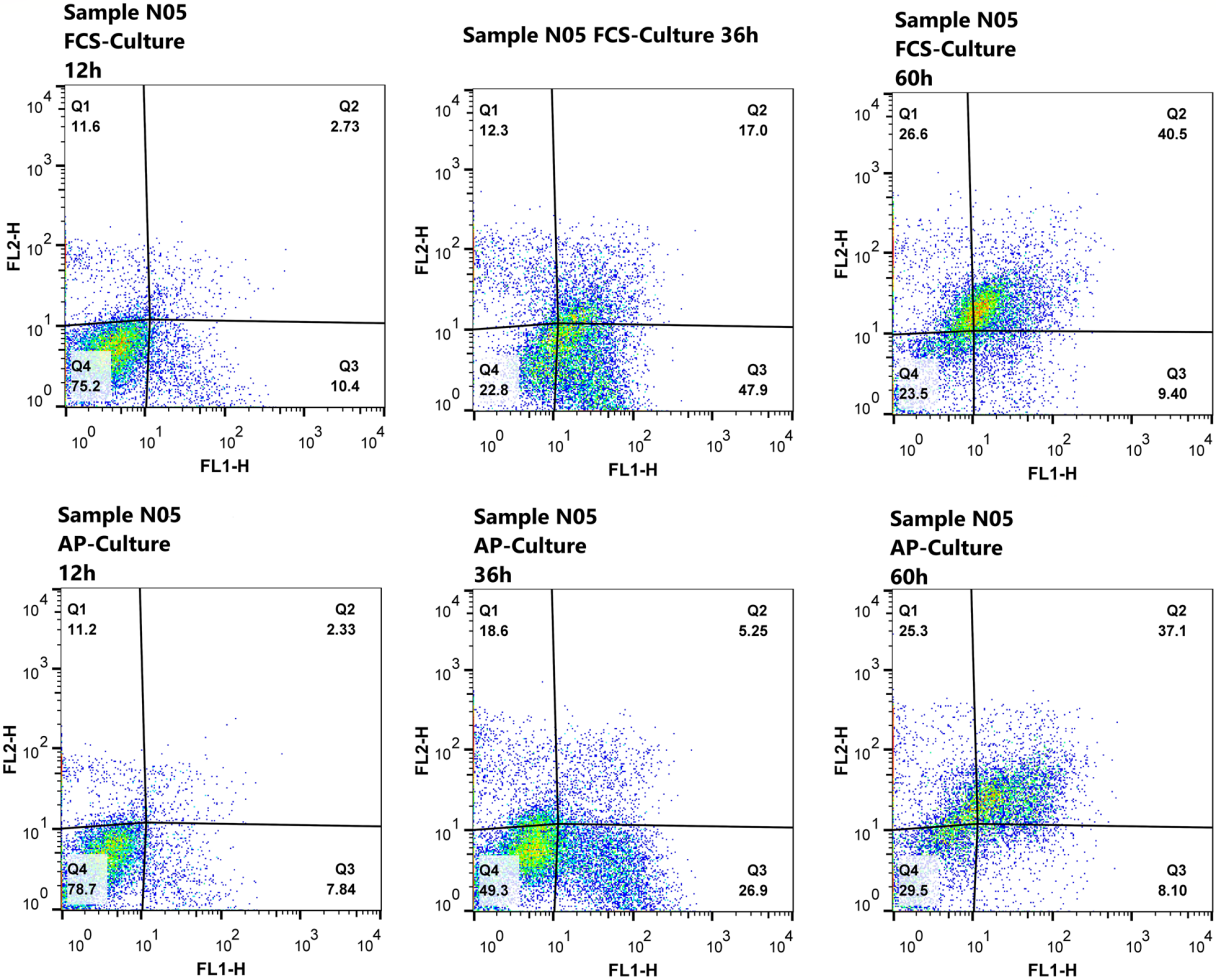
Distinguish of viable and apoptotic neutrophils by flow cytometry. The upper plots show a granulocytes sample in FCS culture after 12 h, 36 h and 60 h respectively. The lower plots are the same sample in AP culture

**Fig. 3:**
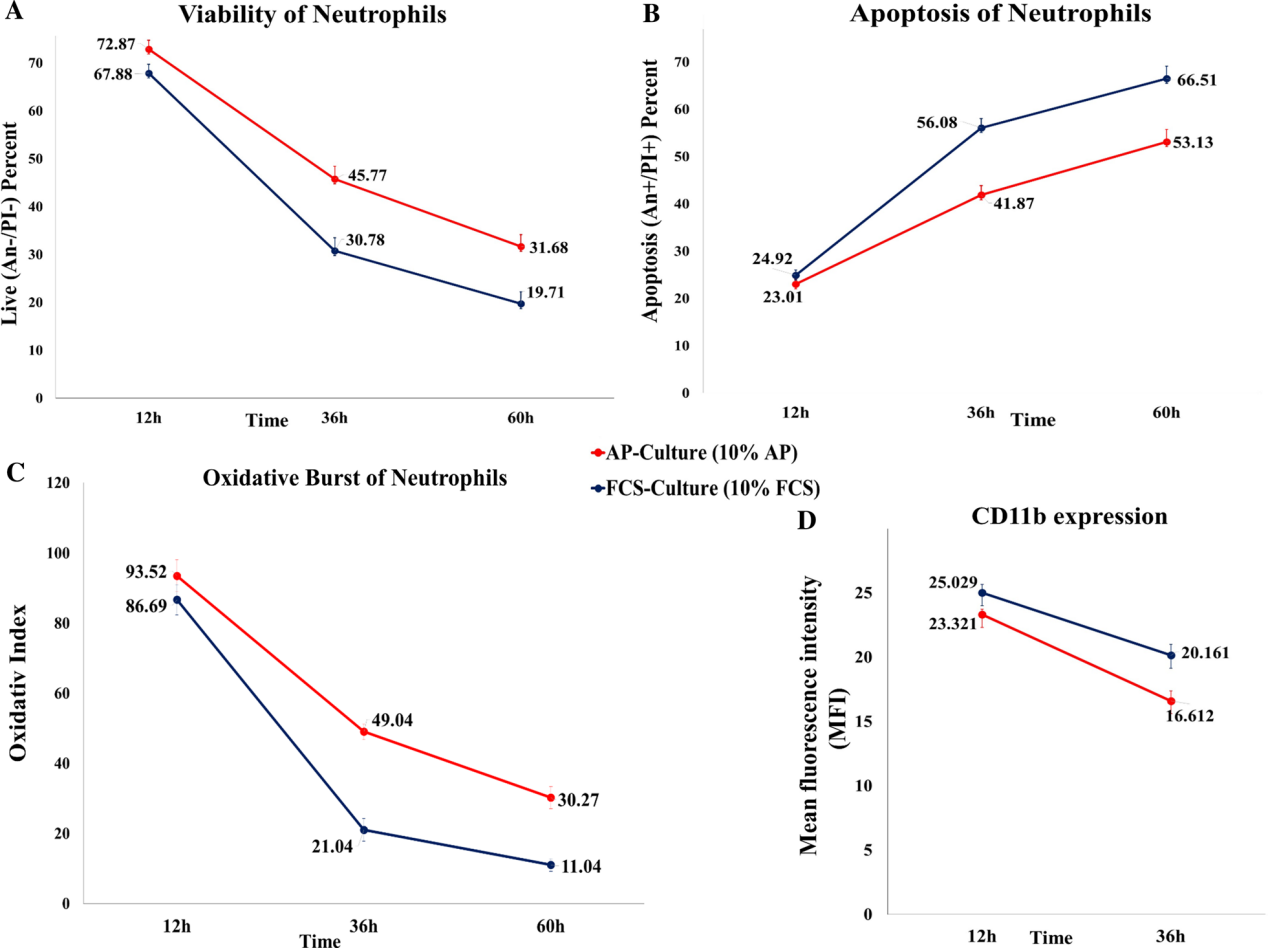
The amount of viable/apoptotic neutrophils, oxidative burst and CD11b expression in AP versus FCS cultures. The rate of apoptosis was inversely proportional to the number of viable neutrophils; the difference between viable (plot A) and apoptotic cells (plot B) were significant at 36 h and 60 h but not at 12 h (n = 32). For comparison of the oxidative burst capacity of neutrophils, “oxidative index” were calculated, that is the ratio of mean fluorescence of stimulated neutrophils to mean fluorescence of unstimulated neutrophils (plot C, n = 28). The increased CD11b expression after LPS stimulation (n = 17) in the neutrophils cultured with two different supplements over time were shown in plot D

Neutrophils were stained by Annexin-V and propidium iodide (PI) to identify apoptotic and viable cells (Fig. 2). The viability of neutrophils in AP cultures and FCS cultures decreased over time. Concurrently, the percentage of apoptotic cells increased in both culture types in a time-dependent manner. Also FCS group showed a more steep reduction in the viability (*P *= 0.003) and a meaningfully higher tendency to undergo apoptosis over the time (*P *< 0.05) (Fig. 3a, b).

### Oxidative burst rate and CD11b expression

No significant differences were observed between AP and FCS cultures (Fig. 3c) (*P *= 0.632) in term of respiratory burst. The levels of oxidative burst did not differ significantly between the two groups at any point in time (*P *= 0.894 at 12 h, *P *= 0.443 at 36 h and *P *= 0.229 at 60 h).

Due to the substantial reduction in the number of neutrophils, it was not possible to assess the CD11b expression at 60 h. There were no significant differences between AP and FCS cultures (Fig. 3d).

The data was further evaluated on the base of the gender of participates (Additional file 2: Figure S1).

## Discussion

Recently, the newly discovered roles of neutrophils in many physiological and pathological conditions has increased in vitro studies on them [14]. The study of neutrophils is relatively difficult because of their sensitivity, inability of proliferation and limited lifespan. The scientific efforts for optimizing the neutrophil isolation and culture have been continued [15, 16]. Currently, blood neutrophils usually are isolated using density-gradient-based methods and cultured in common cell media with FCS. However, a various range of protocols exist for the isolation of neutrophils [16]. Selecting the simplest one that was also economical was key for this study. Thus, regarding the densities of leukocytes [17], a discontinuous two-layer gradient of Percoll was first made; then, this was compared with the current simplest method of neutrophil isolation (the single-step centrifugation on Ficoll). The data showed that using Percoll yields a more homogenous granulocyte population. Contrarily, Grisham et al. [18] reported that neutrophil isolation with Percoll gradients leads to little less purity than Histopaque-isolated neutrophils (Histopaque is a Ficoll-based density medium). None of the studies, found considerable differences, although the main difference between the present protocol and others was the elimination of platelets from the blood before density gradient centrifugation. Whether this change can explain the observed differences, further studies on larger samples are needed, because few samples were evaluated in both studies (n = 5). Concerning our finding and previous reports on the superiority of Percoll over Ficoll for neutrophil separation [19], discontinuous Percoll gradient was used to separate neutrophils from the blood.

FCS is not a proper supplement for cell media. As biologic alternatives, AS and AP have belong to the first proposed options to supplement the cell media. AS is reported to outperform FBS for the cultivation of both human lymphocytes [20] and chondrocytes [21]. AS and AP have been shown to be suitable supplements also for the expansion of various stem cells obtained from different origins without adversely affecting their differentiation capacity [22–25]. However, subsequent studies have turned out that the replacement of FCS by AS or AP is not always effective. Wu et al. [26] observed that for equal cell viability and proliferative ability of human corneal epithelial cell, higher concentration of AS than FBS is needed in the cell culture. Chimenti et al. [13] demonstrated that supplementation of cultures of human cardiac progenitor cells (CPCs) with AS show in a reduced proliferation rate and a shift towards the endothelial phenotype when compared to those obtained with FBS supplementation. Also CPCs displayed a senescent-like morphology with time in culture with AS. Nimura et al. [27], found that compared with FBS, AS decreased the proliferation of bone marrow mesenchymal stem cells (BM-MSC).

Consequently, AS and AP may be assumed as perfect substitutes for xenogeneic FCS, but the successful use of them is remarkably cell-type dependent. The probable differences between AS or AP and FCS have not been determined for neutrophil cell cultures. Here, we compared the viability and function of neutrophils cultured with FCS or AP.

Although compared with plasma, the use of serum is more common, but we chose AP instead of AS to supplement the culture medium because of the following reasons. The components of serum and plasma are similar. Only six proteins out of 80 important tested proteins had a manifold increase in serum rather to the plasma [11]. Of note just one of these factors was a growth factor which has no receptors on neutrophils [28]. All other factors that were higher in serum belong chemokines, which can activate neutrophil chemotaxis and degranulation. This is undesirable because researchers intend to isolate resting neutrophils and maintain them unprimed/inactivated in the culture to be able to investigate them under the condition of interest (such as adding chemicals). Moreover, a recent study demonstrated the equal efficacy of serum and plasma as supplements in primary cell culture (BM-MSC) and also in adherent (HeLa) and suspended cell line cultures (U-937) [29]. Additionally, AP is more available than AS. It can be obtained from the same blood sample that is taken for neutrophil isolation, whereas AS should be extracted from the clotted blood. It also obtains from AP but after an extra calcification step [11]. Besides, serum contains a lot of non-physiologic, serum-specific proteins [30] which may affect sensitive neutrophils. Moreover, in the body, neutrophils are floating in plasma, not serum.

Consistent with previous reports [31], the number of live neutrophils was reduced in a time-dependent manner as a result of apoptosis in either FCS or AP cultures. However, the data showed neutrophil viability was better using AS than FCS, but concerning neutrophil function, no difference could be shown between both groups. This observation may be due to the interspecies differences in the biological/chemical composition of the blood. Alternatively, it is possible that the supporting/survival factors in AP function more efficiently than their bovine equivalents [32]. Obviously these results must be verified in further researches. Although human neutrophils were evaluated in this study, our results may be reproduced with other species such as murine neutrophils.

Additionally, only two main functions of neutrophils were assessed and more studies need to show whether other functions of neutrophils or any other aspect of their biology may be different in AP- versus FCS-supplemented media. However, based on our results AP acts superior to FCS in neutrophil cell cultures. These results may be of value for ever-increasing researches on human (and murine) neutrophils.

## Limitation

A limitation of this technique is related to obtaining AS from PRP. It is possible that remaining platelets cause cell clump in the cell culture if they not be removed from AS completely.

## Supplementary information

**Additional file 1: Table S1.** The demographic characteristics of the subjects. All subjects were Iranian-Persian. The respiratory burst and CD11b analyses could not be done for all samples whose viability was evaluated. “Yes” means that the analysis was performed and “No” means was not performed for the subject.

**Additional file 2: Figure S1.** The neutrophils of men and women behave somewhat differently in AP- and Fcs-supplemented cell cultures. For male, the differences between viable (plot A) and apoptotic cells (plot B) were significant only at 60 h (n = 14) but for female, these differences were not significant only at 12 h (n = 18). The rate of “oxidative index” (plot C) and the increased CD11b expression (plot D) in the neutrophils of two sex subgroups which cultured with two different supplements over time were shown.

## Data Availability

The datasets used during the current study are available from the corresponding author on reasonable request.

## Abbreviations

FCS: fetal calf serum
FBS: fetal bovine serum
AS: autologous serum
AP: autologous plasma
RBCs: red blood cells
PRP: platelet rich plasma
BM-MSC: bone marrow mesenchymal stem cell
CPCs: cardiac progenitor cells
