# Supplementary material for: Autologous plasma versus fetal calf serum as a supplement for the culture of neutrophils
Source: BMC Res Notes. 2020 Jan 22;13:39. doi: 10.1186/s13104-020-4902-z (PMC6977324; doi:10.1186/s13104-020-4902-z)
Supplement: Supplementary file 1 — Additional file 1: Table S1. The demographic characteristics of the subjects. All subjects were Iranian-Persian. The respiratory burst and CD11b analyses could not be done for all samples whose viability was evaluated. “Yes” means that the analysis was performed and “No” means was not performed for the subject. [file 13104_2020_4902_MOESM1_ESM.docx]

**Additional File 1:**

**Characteristics of Subjects.**

In this study the neutrophils obtained from healthy volunteers (aged 25 to 42 years; 14 men and 18 women) who had no current health problems and taken no current medication. The demographic characteristics of the subjects, along with the analysis that was performed for each subject was presented in Table 1. The mean age for women and men were 29 ± 1.11 and 31± 1.35 years, respectively. Note: because of the reduction of neutrophil number during the time, we could not perform respiratory burst and CD11b expression analyses for all 32 samples whose viability was evaluated.

**Table S1. The demographic characteristics of the subjects.** All subjects were Iranian-Persian. The respiratory burst and CD11b analyses could not be done for all samples whose viability was evaluated. “Yes” means that the analysis was performed and “No” means was not performed for the subject.

| Sample Number | Age | Viability  Assessment | Oxidative burst  Analysis | CD11b expression Analysis |
| --- | --- | --- | --- | --- |
| Sample 01 | 29 | Yes | Yes | Yes |
| Sample 02 | 28 | Yes | Yes | Yes |
| Sample 03 | 36 | Yes | Yes | Yes |
| Sample 04 | 25 | Yes | No | No |
| Sample 05 | 37 | Yes | Yes | No |
| Sample 06 | 25 | Yes | Yes | Yes |
| Sample 07 | 30 | Yes | Yes | Yes |
| Sample 08 | 36 | Yes | Yes | Yes |
| Sample 09 | 29 | Yes | Yes | Yes |
| Sample 10 | 30 | Yes | Yes | Yes |
| Sample 11 | 34 | Yes | Yes | Yes |
| Sample 12 | 42 | Yes | Yes | Yes |
| Sample 13 | 32 | Yes | Yes | No |
| Sample 14 | 32 | Yes | Yes | Yes |
| Sample 15 | 29 | Yes | Yes | No |
| Sample 16 | 42 | Yes | No | No |
| Sample 17 | 31 | Yes | Yes | No |
| Sample 18 | 29 | Yes | Yes | No |
| Sample 19 | 31 | Yes | Yes | Yes |
| Sample 20 | 40 | Yes | Yes | Yes |
| Sample 21 | 26 | Yes | Yes | No |
| Sample 22 | 26 | Yes | Yes | Yes |
| Sample 23 | 26 | Yes | Yes | No |
| Sample 24 | 35 | Yes | Yes | No |
| Sample 25 | 41 | Yes | No | No |
| Sample 26 | 38 | Yes | Yes | Yes |
| Sample 27 | 26 | Yes | Yes | No |
| Sample 28 | 27 | Yes | Yes | No |
| Sample 29 | 28 | Yes | Yes | Yes |
| Sample 30 | 27 | Yes | No | No |
| Sample 31 | 27 | Yes | Yes | Yes |
| Sample 32 | 28 | Yes | Yes | No |
