# Supplementary material for: Autologous plasma versus fetal calf serum as a supplement for the culture of neutrophils
Source: BMC Res Notes. 2020 Jan 22;13:39. doi: 10.1186/s13104-020-4902-z (PMC6977324; doi:10.1186/s13104-020-4902-z)
Supplement: Supplementary file 2 — Additional file 2: Figure S1. The neutrophils of men and women behave somewhat differently in AP- and Fcs-supplemented cell cultures. For male, the differences between viable (plot A) and apoptotic cells (plot B) were significant only at 60 h (n = 14) but for female, these differences were not significant only at 12 h (n = 18). The rate of “oxidative index” (plot C) and the increased CD11b expression (plot D) in the neutrophils of two sex subgroups which cultured with two different supplements over time were shown. [file 13104_2020_4902_MOESM2_ESM.docx]

**Additional File 2:**

**The sex-based analysis of data**

We evaluated the result to see whether or not the sex of participates has an effect on the neutrophils viability in FCS and AP cultures. The changes of the apoptosis rate (p=0.018) and the amount of live cells (p=0.022), under effect of the supplementation type (FCS or AP) over the time, was also influenced by the gender of subjects. The cell viability of neutrophils of men and women were better in AP-cultures; however the cells of men were influenced less than women by the culture supplementation (Figure S1A & S1B).

We also found no statistically significant effect of the gender of subjects on oxidative burst rate (p=0.715) as well as on CD11b expression (p=0.875) (Figure S1C & S1D).


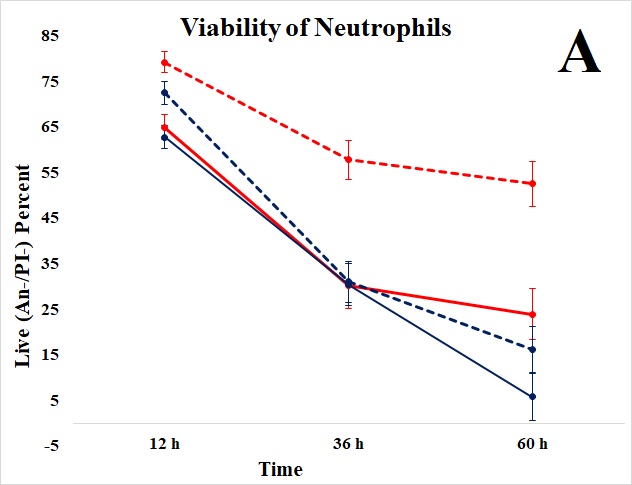

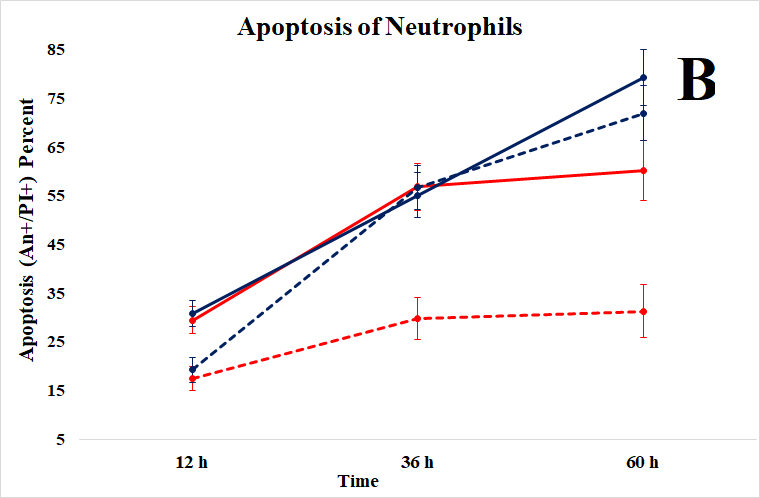


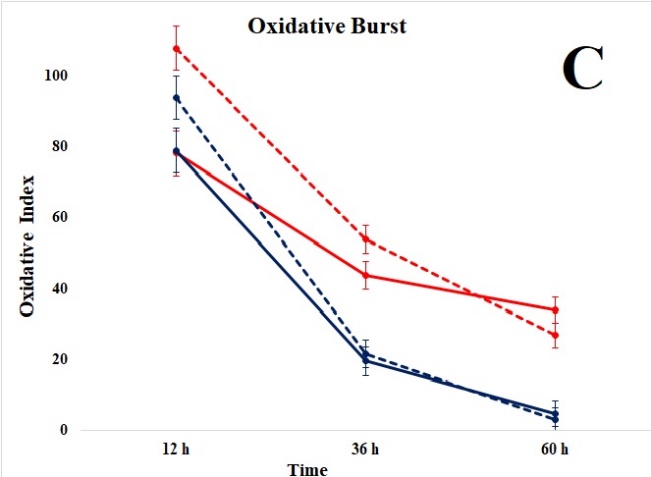

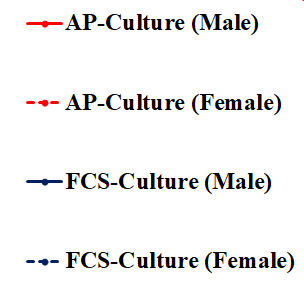

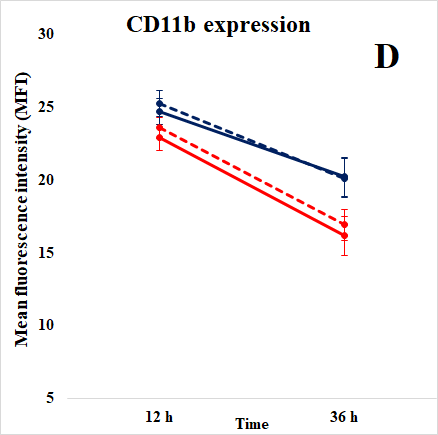


**Figure S1. The neutrophils of men and women behave somewhat differently in AP- and FCS-supplemented cell cultures.** For male, the differences between viable (plot A) and apoptotic cells (plot B) were significant only at 60 h (n=14) but for female, these differences were not significant only at 12 h (n=18). The rate of “oxidative index” (plot C) and the increased CD11b expression (plot D) in the neutrophils of two sex subgroups which cultured with two different supplementations over time were shown.
